# Theoretical foundation for real‐time prostate localization using an inductively coupled transmitter and a superconducting quantum interference device (SQUID) magnetometer system

**DOI:** 10.1120/jacmp.v5i4.2021

**Published:** 2004-11-24

**Authors:** John. E. McGary

**Affiliations:** ^1^ Department of Radiology Baylor College of Medicine Houston Texas 77030 U.S.A.

**Keywords:** localization, transponder, transmitter, prostate localization

## Abstract

Real‐time, 3D localization of the prostate for intensity‐modulated radiotherapy can be accomplished with passively charged radio frequency transmitters and superconducting quantum interference device (SQUID) magnetometers. The overall system design consists of an external dipole antenna as a power source for charging a microchip implant transmitter and SQUID magnetometers for signal detection. An external dipole antenna charges an on‐chip capacitor through inductive coupling in the near field region through a small implant inductor. The charge and discharge sequence between the external antenna and the implant circuit can be defined by half duplex, full duplex, or sequential operations. The resulting implant discharge current creates an alternating magnetic field through the inductor. The field is detected by the surrounding magnetometers, and the location of the implant transmitter can be calculated. Problems associated with this system design are interrelated with the signal strength at the detectors, detector sensitivity, and charge time of the implant capacitor. The physical parameters required for optimizing the system for real‐time applications are the operating frequency, implant inductance and capacitance, the external dipole current and loop radius, the detector distance, and mutual inductance. Consequently, the sequential operating mode is the best choice for real‐time localization for constraints requiring positioning within 1 s due to the mutual inductance and detector sensitivity. We present the theoretical foundation for designing a real‐time, 3D prostate localization system including the associated physical parameters and demonstrate the feasibility and physical limitations for such a system.

PACS number: 87.53.‐j

## I. INTRODUCTION

The goal of intensity‐modulated radiotherapy (IMRT) is to minimize the dose to normal tissue surrounding the clinical target volume (CTV) and increase the tumor dose where possible. Any deviations between the planned and treated position degrade the therapeutic ratio. The planning target volume is defined to include the CTV and associated treatment uncertainties that include but are not limited to patient setup and organ motion. For conditions where planning margins are not adequate, the tumor will be underdosed. In contrast, margins that are too large may lead to greater complications. While it is impossible to eliminate these errors, the goal in localization is to reduce the uncertainties where possible.

Prostate motion is a major factor contributing to treatment uncertainty, either from internal movements due to breathing, patient movement during treatment, or from daily variations. With respect to motion as defined by a positional change of the prostate at the time of treatment relative to the planning position, a number of studies evaluated prostate displacements for different conditions and methods.^(^ ^1^ ^–^ ^7^ ^)^ Radio‐opaque markers, gold seeds, CT‐CT fusion, and CT chamfer matching are examples of methods used to determine the prostate position relative to fixed bony landmarks. Within these studies, values of anterior‐posterior (A/P) shifts range from –0.9mm mean, 1.7 mm standard deviation to –5.4mm mean, 6.2 mm standard deviation. The range in the superior‐inferior (S/I) direction was −0.2mm mean, 3.2 mm standard deviation to –5.9mm mean, 5.0 mm standard deviation. The lateral displacements were shown to be minimal: the mean and standard deviations were both less than 1.5 mm.

Along with prostate motion studies, patient setup accuracy was investigated to establish margins for planning target volumes.^(^ ^8^ ^–^ ^13^ ^)^ These studies typically showed that treatment accuracy was increased by immobilization techniques and setup correction strategies.^(^ ^14^ ^–^ ^17^ ^)^ From these studies, the standard deviation of setup errors for prostate patients was reported to range from 2.1 mm to 3.8 mm, 2.5 mm to 3.0 mm, 2.5 mm to 5.5 mm for the lateral, S/I, A/P directions.^(^ ^11^ ^–^ ^14^ ^,^ ^17^ ^)^ Most of the analysis was based on daily portal films or electronic portal images, although some authors retrospectively analyzed their weekly port films. A current summary of setup errors and organ motion results is found in Antolak et al.^(^ ^7^ ^)^

In our clinical application, we developed an immobilization/localization system with an offline correction protocol, specifically for prostate IMRT using the NOMOS Peacock system (NOMOS Corporation, Cranberry Township, PA).^(^ ^18^ ^)^ The system design consists of a Styrofoam pellet filled vacuum bag (Vac‐Lok™ bag, MED‐TEC, Orange City, IO) attached to a carrier box with specialized fiducials for pre‐ and posttreatment localization verification using lateral portal films. In addition to skeletal immobilization, we implemented a system to immobilize the prostate organ using a rectal balloon.^(^ ^19^ ^)^ From our studies, we determined that the standard deviations of setup error were 5.23 mm in the S/I direction, 2 mm in the lateral direction, and 2 mm in the A/P direction. Using the rectal balloon, the prostate was relatively immobilized with motions less than 1 mm in the A/P and lateral directions. The major displacements were observed in the S/I direction, where the standard deviation was 1.8 mm. In addition to daily reproducibility, intra‐treatment motion due to breathing was measured and found to be insignificant. With this system, the current treatment‐planning margin is 12.5 mm in the S/I direction and 5 mm in the axial plane, which limits our mean prostate dose to ~77Gy.^(^ ^20^ ^,^ ^21^ ^)^ This system is far from ideal toward developing further dose escalation.

Solutions to tumor localization have been investigated using electronic portal imaging devices (EPIDs) for more than a decade.^(^ ^22^ ^–^ ^26^ ^)^ With respect to setup verification, posttreatment analysis has been used to estimate setup errors defined by bony landmarks.^(^ ^11^ ^,^ ^16^ ^,^ ^27^ ^–^ ^31^ ^)^ Planar images compared with the original 2D X‐ray image, either by visual inspection or simple analysis, was used to determine the setup distribution for a group of prostate patients. For on‐line setup correction, protocols have been implemented at several institutions over the last decade.^(^ ^23^ ^–^ ^32^ ^,^ ^33^ ^)^ Early strategies used visual inspection of pretreatment ports and subsequent patient correction. With this strategy, corrections were based on subjective visual interpretations, images provided only 2D views, and patient setup time was long. Even though patient setups were corrected before treatment, the posttreatment analysis discovered that the setup errors were larger than believed at the time of treatment, which was between 5 mm and 10 mm. Due to these problems and the expense, very few centers used this method for setup correction. The technology is improving; however, automatic correction systems do not yet exist for EPID technology.

Another localization device implemented for clinical treatment is the BAT (B‐mode Acquisition and Targeting) ultrasound system (NOMOS Corporation). BAT was developed to visualize the prostate organ before treatment.^(^ ^34^ ^)^ This system requires that a technologist visually compare images received from the transducer array with a reference image to judge the prostate displacement error. Depending upon the protocol defined at each institution, the patient is moved before treatment to reduce setup errors.^(^ ^35^ ^)^ Similar to the flaws inherent with EPID technology, ultrasound accuracy is limited by the lack of quantitative tools, 2D data, and the specific technologist skill level. The setup uncertainty using this system is greater than 5 mm and does not correct for intra‐treatment prostate motion that can be larger than 10 mm for total displacement.^(^ ^36^ ^)^

Currently, traditional imaging methods are 2D acquisitions, and multiple images are required to generate a 3D reconstructed image volume. The reconstructed images need to be further processed to calculate the relative displacement from the planning image set, which is time‐consuming and subject to several errors. One error is that the tumor can move during the time that the images are collected or processed, either during image acquisition or during the 3D displacement calculation. Another contribution to the overall displacement uncertainty results from the algorithm used to calculate the displacement. With these problems, traditional imaging systems are currently unable to provide accurate, real‐time organ location needed for real‐time correction or posttreatment analysis.

Ideally, a localization system would transmit an accurate, real‐time, 3D position that would allow real‐time corrections during treatment or posttreatment adaptive radiotherapy. A simple system with these properties may exist through the use of radio frequency transmitters or transponders. As an example, a wireless transponder system has recently been developed for localization using a transponder system and is currently being tested.^(^ ^37^ ^)^ However, the details of the system have not been published, and the fundamental physical principles or theory have not been disclosed.

To understand how a transmitter system might work for prostate localization, we will investigate the basic physical principles underlying the process and determine the necessary conditions required for operation in a heuristic manner. With these concepts presented here, the feasibility of designing a transmitter system in terms of signal strength and real‐time capability will be examined. Signal generation, in terms of mode, strength, and detection will be the focus of this discussion.

## II. METHODS AND MATERIALS

The proposed concept is to develop a remote localization system that will transmit an electromagnetic signal from an electronic device implanted within a tumor; the implant position will be calculated using the detected signal at different locations. Within the general concept of signal generation and detection, there are two methods that might be applicable: (1) using the magnetic, electric, or coupled field measurements to calculate the location; or (2) using the time of flight for a signal to travel between the source and detector. The second method requires more accurate timing measurements than currently possible for 1‐mm resolution. Therefore, the first method will be explored as the initial approach.

There may be a variety of methods for signal transmission and detection using the electric, magnetic, or coupled fields; however, we have chosen the magnetic induction vector as the principle source due to several design constraints illustrated in the following section. Within the category of generating and detecting a magnetic field, the time variation of the field strength can be static or dynamic. To examine a simple case of using a static field, a small magnetized bar magnet could be implanted within the tumor; the associated magnetic field strength could then be used to determine the location. The physical properties associated with generating and detecting the field strength are the detector sensitivity, the magnetic field strength, and the implant size. With respect to implant size, the size should be comparable to brachytherapy seed implants. Therefore, an estimate for the size of the magnet dimensions is on the order of 5mm diameter×5mm length. Considering these dimensions, the magnetic dipole moment for a permanent magnet is easily calculated using

(1) $$M=\frac{B_{i}V}{\mu_{0}}$$

where *M* is the magnetic dipole moment (A·m^2^), $B_{i}$ is the intrinsic magnetic induction of the permanent magnet (T), *V* is the volume (m^3^), and $\mu_{0}$ is the permeability constant, $\mu_{0}=4п\times 10^{-7}\;H/m$. Using typical values for the magnetic induction, $B_{i}\sim 0.5\;T$ for ceramic or ferrite magnets and the magnet dimensions, $V=1.25\times 10^{-7}m^{3}$, the calculated magnetic moment, *M,* is approximately 0.05 A m^2^. With these values, the magnitude of the magnetic induction field at large distances, along the direction of the magnetic moment, is calculated using the magnetic dipole equation:

(2) $$B\approx \frac{\mu_{0}M}{4\pi \;r^{3}}$$

where the approximation is used for distances much larger than the permanent magnet dimensions.

At a distance of 1 m from the permanent magnet, B~5nT, which can be measured using conventional magnetometers in magnetically quiet environments. However, if implant position accuracy is to be resolved within 2 mm, the detectors will be required to measure induction signals in the picotesla range, which is insufficient for detection using conventional magnetometers except in magnetically shielded rooms. Table 1 shows estimates in the variation of *B*(*r*) as a function of distance between the point of measurement and the magnet, where dB(r)~(−3B(r)/r)dr. The third column, ΔB, shows the variation in *B*(*r*) over a 2‐mm distance, indicating the measurement values required to resolve 2 mm positional accuracy. As the detection distance from the magnet decreases, the magnetic induction increases by $r^{3}$, which reduces the sensitivity needed for the detectors. However, ΔB continues to be relatively small even at a distance of 50 cm, which is important for clinical applications.

**Table 1:** The magnetic field variation with distance for a permanent magnet

*r* (m)	*B*(*r*) (nT)	ΔB(r+2mm)(nT)
1.0	5.0	~−0.03
0.50	40.0	~−0.5
0.10	5000.0	~−300

*r* is the distance from the magnet center

*B(r)* is the magnetic induction

B(r+0.002) is the magnetic induction at 2 mm displacement from the origin.

Acknowledging that the magnetic flux density from a permanent magnet is relatively small compared with the radio frequency noise generated from the LINAC components, another method for generating a magnetic field is needed. Pulsing the induction field will allow detectors to discriminate the desired signal from other static and oscillating magnetic fields within the treatment room. With a defined generating frequency, the associated detectors can selectively filter out the background fields and measure the relatively small magnetic field strength. A well‐known method for creating a simple, pulsed magnetic induction field is by generating an alternating current in a conductor loop. The magnetic field intensity of an oscillating dipole magnetic field can be calculated analytically for distances large compared with the coil radius as follows:

(3) $$H\approx \left(\frac{m_{0}}{4\pi r\lambda^{r2}}\right)\text{exp}(j\omega t)\left[2\left(\frac{\lambda^{r2}}{r^{2}}+\frac{\lambda^{t}}{r}j\right)\text{cos}\theta \hat{r}+\left(\frac{\lambda^{r2}}{r^{2}}+\frac{\lambda^{t}}{r}j‐1\right)\text{sin}\theta \hat{\theta }\right]$$

where *H* is the magnetic field intensity (A/m), $\lambda^{\prime }=п\lambda$ is the wavelength (m) of the alternating current loop, $\omega \;\text{is the angular frequency}=2пf(s^{-1})$, *r* is the radius vector to point of measurement, and $m_{0}$ is the magnetic moment of the current loop given by

(4) $$m_{0}=\frac{1}{2}⨖I(r^{\prime })r^{\prime }\cdot dl^{\prime }$$

where *I* is the current.^(^ ^38^ ^)^ There are several extreme conditions of analytic interest: for distances that are much larger than the wavelength of the pulse, r>>λ, the field intensity is dependent upon the frequency and inversely proportional to the distance:

(5) $$H=\left(\frac{‐m_{0}}{4\pi r\lambda^{t2}}\right)\text{exp}\;(j\;\omega \;t)\left[\text{sin}\;\theta \;\hat{\theta }\right]$$

and for conditions where the frequency is zero, the magnetic field intensity reduces to the static dipole equation:

(6) $$H=\left(\frac{m_{0}}{4\pi r^{3}}\right)\left[2\cos\theta \hat{r}+\sin\;\theta \hat{\theta }\right]$$

For all conditions where the distance is large with respect to the coil radius, the magnetic field intensity can be calculated using Eq. (3) at any point in space relative to the center of the loop.

There are a number of factors to consider with regard to this type of design using an oscillating magnetic dipole to generate a detection signal: (1) the operating frequency, (2) detector distance, (3) energy source, and (4) size of the device. All of these factors are mutually dependent and require a balance to meet the basic design constraints. The operating frequency for pulsing the current loop is important with respect to transmission. At detection distances much greater than the wavelength associated with the operating frequency, the oscillating dipole is viewed as a radiator or magnetic dipole antenna where the magnetic and electric fields detach from the current source and radiate into space as electromagnetic waves. This is known as the far field region, and Eq. (5) is applied in that region. The transition reference distance between the far and near field is described by $R_{F}=\lambda /2п$, where RF is the transition range. Table 2 shows $R_{F}$ for several operating frequencies.

**Table 2:** Induction field range as a function of frequency

Frequency (kHz)	Wavelength (m)	RF (m)
<135	2222	353
$6.78\times 10^{3}$	44.7	7.1
$13.56\times 10^{3}$	22.1	3.5

RF = the transition range separating the near and far field regions

Within the far field region, the electromagnetic energy dissipation is a function of frequency. The energy density for conductors is described by

(7) $$S_{mg}=0.5\sqrt{\left(\frac{\sigma }{2\omega \mu }\right)}\text{exp}(‐2zf\delta )E_{0}^{2}$$

where *z* is the propagation distance, δ is the skin depth, ω is the angular frequency, σ is the conductivity, and μ is the permeability of the medium. Dissipation within a conducting medium is dependent upon the square root of the frequency *f*); higher frequencies are attenuated more than lower frequencies. For tissue, the energy attenuation is approximately −7.96dB/10cm at 10 MHz and decreases to approximately −0.252dB/10cm at 1 kHz.^(^ ^39^ ^)^ At the higher operating frequencies, two problems arise: (1) the surrounding medium has to be known in advance to calculate the attenuation and the induction field for determining the location, and (2) the signal magnitude is reduced at the detector, making accurate measurements more difficult. In contrast, if the operating frequency is within the low‐frequency range, 50 kHz to 200 kHz, attenuation problems are essentially eliminated and Eq. (3) can be used without corrections. In addition, the transition distance, RF, is greater than ~300m, which is large compared with the dimensions of a radiation therapy room and allows detection to be within the near field region. For these reasons, the transmission frequency should be chosen to be within the low‐frequency range.

Providing power to the alternating current loop is a major problem that depends upon the operating frequency and other factors. Presently, small batteries exist that can be used to generate the current needed to create the magnetic field strength but require physical space and increase the overall dimensions of the implant. In addition, batteries within an implant pose extra problems relating to health regulations, which may be more difficult than the size constraints of the device. Since the concept of an active power source is not desirable at this time, the design should incorporate a passive technique. To address this problem, the coil used for generating the detection signal can be used as the same conductor loop to simultaneously act as an inductor for energy transfer. Therefore, including a capacitor into the circuit design provides a method to store energy from an external inductor power source. To expand upon this, we consider the system as two conductor loops; one loop or coil is part of the implant device (loop 2), and the other coil belongs to the external power source (loop 1), where the two conductor loops are relatively close to each other. The two circuits are then connected by the partial flux, which depends upon the relative geometry and position of the two conductor loops. The mutual inductance of loop 2 from loop 1 is defined as the ratio of the partial flux enclosed by the second conductor loop to the current in the first loop and is expressed as

(8) $$M_{21}=\underset{A_{0}}{⨖}\frac{\bar{B_{2}(l_{1})}}{l_{1}}•\bar{dA_{2}}$$

with $M_{21}=M_{12}=M$.

With the two conductors coupled, the second conductor loop will generate an electric field due to the change in magnetic flux from the oscillating current in loop 1 (Faraday's law) and induce a voltage across the terminals of an open loop circuit:

(9) $$•\frac{\partial \Phi }{\partial \tau }=⨖\bar{E}•\bar{ds}$$

This is the basis for supplying power to the implant circuit using a low‐frequency alternating magnetic field, which reduces to a simple transformer circuit when operated within the near field region. Figure 2 shows the two current loops and their associated components, demonstrating the basic configuration in terms of circuits.

The equation for this circuit is well known and is described by a simple second‐order differential equation:

(10) $$L_{2}\frac{d^{2}Q_{2}(t)}{dt^{2}}+R_{2}\frac{dQ_{2}(t)}{dt}+\frac{Q_{2}(t)}{C_{2}}=M_{12}\frac{dl_{1}(t)}{dt}$$

where the charge in the implant circuit can be calculated with respect to time. This represents the basic electronic circuit for passive charging where charge can be accumulated using rectifiers in several ways. The first possibility would allow the charge to be accumulated over time, which can be classified as sequential charging. In this mode, charge can be collected until the designed operating potential is achieved, and then the collected charge would be discharged through a signal generation circuit. Another possibility exists where the circuit would continuously charge and discharge in relation to the antenna frequency. In that case, the operating mode would be classified as full or half duplex. In this mode, the transmitter can generate a signal without interruption as long as the power source is activated. For this case, the transmission circuit can be represented by the load resistance, $R_{L}$ (Fig. 3).

Since we are interested in current and voltage, the current measured across the load resistance is given by $u_{2}/R_{2}$. The induced current in loop 2 generates an additional time‐varying magnetic flux that opposes the flux from loop 1. The circuit can be summarized with the following equation:

(11) $$u_{2}=\frac{\partial \Phi_{2}}{\partial t}=M\frac{di_{1}}{dt}‐L_{2}\frac{di_{2}}{dt}‐i_{2}R_{2}$$

Alternatively, the equation can be rewritten in terms of $i_{1}$ as a sinusoidal current by

(12) $$u_{2}=\frac{j\omega M\;i_{1}}{1+\frac{j\omega L_{2}+R_{2}}{R_{L}}}$$

For conditions where the load resistance is large, $R_{L}\to \infty$, the voltage depends upon the frequency, mutual inductance, and current in loop 1, $u_{2}=j\omega {\text{Mi}}_{1}$. In the case where the additional circuit load approaches zero, the induced voltage approaches zero $\left(R_{L}\to \gamma 0,\;u_{2}\to \gamma 0\right)$. This demonstrates that the operating characteristics of the external circuitry are related to the power transmission frequency and inductance: higher antenna frequencies generate more induction current and load voltage. This is part of the design compromise, since higher frequencies deliver more current but are also attenuated more through tissue, and the operating frequency requires some degree of optimization.

Since coupling between the power coil (antenna) and the signal generation loop is small, the induced voltage at $R_{L}$ must be maximized. This is accomplished using a resonant circuit design where the basic circuit is represented by that in Fig. 4. The circuit consists of an induced voltage at the inductor and a design capacitor $C_{2}$, which is the combination of parasitic capacitance $\left(C_{P}\right)$ and a parallel capacitor $\left(C_{2}^{\prime }\right)$. The resonant frequency of the parallel resonant circuit can be calculated using Thomson's equation:

(13) $$f=\frac{1}{2\pi \sqrt{L_{2}C_{2}}}$$

The voltage at the load resistor for this resonant circuit is

(14) $$u_{2}=\frac{j\omega M\;i_{1}}{1+(j\omega \;L_{2}+R_{2})\left(j\omega \;C_{2}+1{/}_{R_{L}}\right)}$$

The circuit can be designed to step up the voltage for weak coupling. However, the step‐up voltage is difficult to calculate due to the mutual inductance dependence on geometry and distance.

With uncertainties in mutual inductance due to variations in geometry and distance, a shunt resistor or a parallel regulator can be inserted to maintain the voltage required for the operational characteristics of the support chip. This is necessary since the voltage step‐up could vary by two orders of magnitude depending upon the frequency, distance, and orientation of the device relative to the power inductor. In the basic circuit description in Fig. 4, the regulator is designed as an electronic circuit in parallel with the load resistance such that the internal resistance falls abruptly when the threshold voltage is exceeded. In this configuration, power can be transmitted continuously to the signal generating circuit without interruption as long as the mutual inductance, frequency, and operating voltage are matched.

## III. RESULTS

With the design constraints defined as low‐frequency operation in the near field region, the concept for energy transfer from an external magnetic field to the implant circuit is by induction. To provide power to the transmitter, a large inductor coil with an alternating magnetic field can be placed within the range of the implant coil. In addition to creating an induction field in the near field region, the power transfer circuit is also considered to be an antenna at large distances, as shown earlier. There are several points to consider for this type of configuration design: (1) the minimum magnetic field strength as a function of distance required to power the implant circuit; (2) the antenna current loop radius; (3) the operating frequency; and (4) the associated licensing regulations governing antennas.

**Figure 1:**
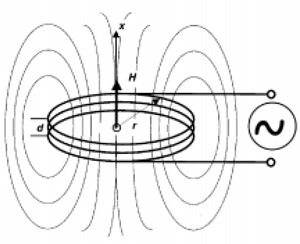
Oscillating magnetic dipole

To estimate some of the properties associated with the power antenna design, Eq. (15) below can be used to calculate the magnetic field intensity along the *x*‐axis of the coil (Fig. 1):

(15) $$H=\frac{1NR^{2}}{2\sqrt{{\left(R^{2}+x^{2}\right)}^{3}}}$$

where *R* is the radius of the antenna coil, and *x* is the distance along the normal axis to the coil plane.

**Figure 2:**
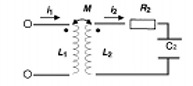
Inductor loop 1 transfers energy to inductor loop 2, and the energy is stored in capacitor *C*2

**Figure 3:**
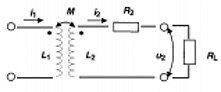
Continuous energy transfer to inductor loop 2

**Figure 4:**
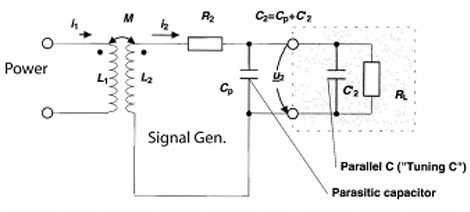
Field strength as a function of radius and distance. The current and number of windings are the same for all calculations.

**Figure 5:**
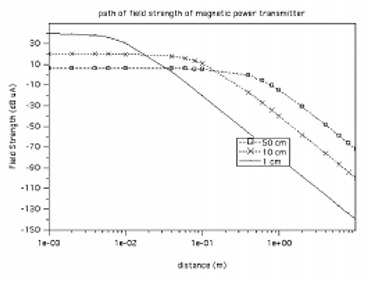
Field strength as a function of radius and distance. The current and number of windings are the same for all calculations.

For a slightly more complicated rectangular conductor loop with side lengths *a* and *b*, Eq. (16) below can be used for better estimates:

(16) $$u_{2}=\frac{j\omega \mu_{0}H_{\text{eff}}AN}{1+(j\omega L_{2}+R_{2})(\frac{1}{R_{L}}+j\omega C_{2})}$$

Figure 5 shows the magnetic field strength, *H*(*x*), of a dipole antenna for three different radii: R=1cm, R=10cm, and R=50cm. The number of windings and the current are the same for each antenna, N=1 and I=1 A. These simple calculations demonstrate that the field strength is relatively flat for distances less than the antenna coil radius (x<R), and then it decreases sharply for distances larger than the radius. For the smallest antenna diameter, the magnetic field strength at the coil center is greater than that generated by the larger radii antennas. At larger distances, the converse is true where the smaller antennas exhibit field strengths that are much less than the larger antennas. Although not shown here, the dipole antenna magnetic field intensity decreases rapidly in the near field region, 60 dB per decade, and then decreases more slowly in the far field region, 20 dB per decade.

**Figure 6:**
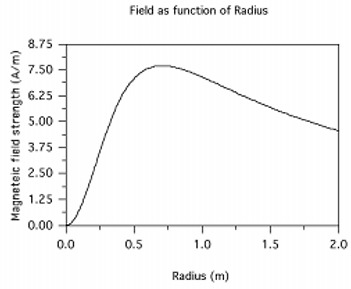
The field intensity *H* at x=0.5m as a function of antenna radius (*R*)

Another relation between magnetic field strength and antenna radius is that the maximum intensity along the axis is found to be at a distance that is roughly equal to the antenna radius. Differentiating Eq. (15) and setting it equal to zero provides the relation $X_{m}=Rv2$, where $X_{m}$ is the distance of maximum field strength along the *x*‐axis. Figure 6 demonstrates the variation in field strength at a fixed distance of 0.5 m while the antenna radius is varied from 0 m to 2 m. This indicates that the maximum intensity is generated with an antenna radius ~0.5v2. For practical design estimates, the field intensity can be considered to have a maximum value at distances that are approximately equal to the antenna radius.

**Figure 7:**
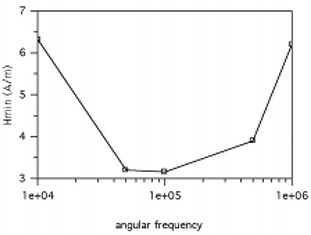
The minimum magnetic field strength required to create a 10 mV operating potential as a function of frequency

With respect to the implant circuit, it is useful to estimate the minimum magnetic field intensity that is just sufficient to supply enough energy to operate the additional support circuitry at the maximum distance from the implant. To find the minimum field strength, we can use Eq. (14) and note that the inductive voltage of the transmitter is $u_{i}=\mu_{0}A\;N\omega H_{\text{eff}}$, where *A* is the area of the coil, *N* is the number of windings, ω is the angular frequency of the dipole antenna source, and $H_{\text{eff}}$ is the antenna field strength at the transmitter coil. With these two equations, the chip operational voltage is

(17) $$u_{2}=\frac{j\omega \mu_{0}H_{\text{eff}}AN}{1+(j\omega L_{2}+R_{2})(\frac{1}{R_{L}}+j\omega C_{2})}$$

and solving for $H_{\text{eff}}$ and setting as $H_{\text{min}}$ to be the minimum field strength at which there is enough energy for the chip.

Figure 7 shows that the minimum field strength corresponds to the resonant frequency of the LC implant circuit. Therefore, the optimal configuration is to set the dipole antenna frequency to the resonant frequency of the transmitter. Again, this dictates further optimization in terms of the implant circuit design, which influences the power antenna design. In Fig. 7, we have selected reasonable approximations for the implant device for the expected operating conditions: $N_{2}=10$, $L_{2}=10^{-5}\;H$, $C_{2}=10^{-5}\;F$, $R_{2}=10^{3}\Omega$, $R_{L}=10\Omega$, coil radius=0.002m, $u_{2}=0.01\;V$, and $\mu_{r}=2500$. We have slightly modified the equations by including the use of a ferrite rod within the implant coil to increase the magnetic induction field to improve coupling and increase inductance. Without the additional ferrite, the required minimum magnetic field to operate the circuit is much greater than legally permitted. From these values, we find that the magnetic field strength required to power the transmitter is too large with respect to regulated values. However, for illustration, we chose an operating potential for the circuit as 10 mV, which is far too small for practical chip design. Without substantial improvements in the circuit parameters, such as the permeability of the inductor core or loop radius, the magnetic field strength will be larger than allowable, which demonstrates the difficulty in designing a full or half duplex circuit of these dimensions.

**Figure 8:**
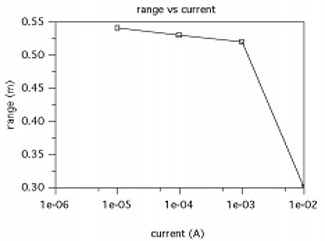
The range as a function of current (power) for an operating potential of 10 mV using the circuit elements used in with I=10A, N=1, and R=0.5m

Another factor for consideration is to determine the maximum range between the antenna and the implant circuit such that the antenna can operate the transmitter circuit with the required chip power. To estimate this range, Eq. (15) can be solved in terms of *x* using $H_{\text{min}}$ from Eq. (17), which is given by

(18) $$x_{\text{range}}=\sqrt{3\sqrt{{\left(\frac{INR^{2}}{2H_{\text{min}}}\right)}^{2}}‐R^{2}}$$

The range is plotted as a function of current to estimate the power needed to operate the chip at 10 mV. We have used the same circuit elements for estimating the minimum field strength in Fig. 7 except for the variation in $R_{L}$. The results shown in Fig. 8 demonstrate that the range is limited to about 50 cm for very low chip power requirements, where the current is less than ~1mA. For higher power requirements, the energy range decreases rapidly toward zero. For practical purposes, the basic circuit cannot be used for half or full duplex operation, since most chip designs require about 1 V to 2 V and roughly 1 mW for power. With the implant design requirements of low frequency and small coil radius, the external magnetic field required to continuously power the circuit would be significantly larger, by a factor of 100, than allowed by licensing regulations. Therefore, the operating mode of the system should be designed to sequentially charge and discharge the main capacitor to generate the necessary magnetic field.

**Figure 9:**
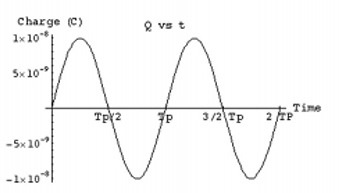
Charge on implant capacitor generated from external induction field. $C_{2}=10^{-5}\;F$, $L_{2}=10^{-5}\;H$, $R_{2}=10^{2}\Omega$, $\omega =10^{5}\;s^{-1}$, $I_{1}=10\;A$, $M_{12}=10^{-7}\;H$.

**Figure 10:**
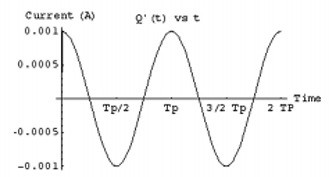
Current on implant inductor generated from external induction field

**Figure 11:**
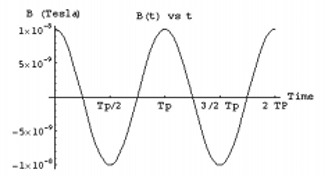
Magnetic induction field at 1 m along the axis generated from current induced into implant inductor

Two major design problems are associated with the sequential operation of the circuit: (1) the amount of charge needed to generate the required magnetic field, and (2) the time to charge the capacitor. To estimate these parameters, the equation representing Fig. 2 (Eq. (10)) can be solved using the circuit values from the previous discussion, where the external alternating current from the power loop is assumed to be a simple sinusoidal current. The charge on the capacitor, shown in Fig. 9, peaks at $\sim 10^{-8}\;C$ with an inductor current of $\sim 10^{-3}\;A$ (Fig. 10). The resulting magnetic induction field can be calculated along the solenoid axis at 1 m to approximate the signal magnitude, as demonstrated in Fig. 11. The resultant field strength generated continuously from this type of circuit can be detected using superconducting quantum interference magnetometers (SQUID), since the detection level extends down to the picotesla level, which is necessary for millimeter accuracy at this level. In practice, however, the field strength may be smaller than these estimates due to the mutual inductance uncertainties, and the accuracy would decrease. To solve this problem, the current can be integrated over time and then discharged later with a larger current than without integration. For this example, integrating over 1 s, which is about 1000 periods, will accumulate about $5\times 10^{-5}\;C$. Ideally, the collected charge could be discharged over 10 to 100 periods and increase the field strength by a factor of 10 or more.

## IV. DISCUSSION

Prostate displacement error as defined by any positional change at the time of treatment relative to the planning position includes skeletal and organ displacements. These can either be static or dynamic. Static displacements can be regarded as positional changes relative to the treatment‐planning position where the prostate and skeleton remain motionless during treatment; dynamic displacements relate to motion during treatment. Dynamic prostate motion is attributed to breathing and skeletal motion. Breathing affects the prostate either from internal motion or due to skeletal motion during deep breathing. In addition, the patient may move during treatment as a result of discomfort. Depending upon the treatment setup, prone or supine, the internal organ displacement magnitudes are different. For systems using an organ immobilizer such as a rectal balloon. the dynamic motion is greatly reduced. The organ displacement relative to the planning position is small, between 1 mm and 4 mm, and the motion during treatment is about 1 mm, except for deep breathing and patient skeletal motion. Even with precise setup, where the prostate is exactly in the same position used for treatment, the patient may move or the prostate may undergo motion due to deep breathing. The time scales for these types of motion are much greater than nonimmobilized prostate treatment.

Since the time scales for breathing are on the order of seconds, the dynamic motion of the prostate is on the order of seconds for real‐time localization. In situations where the prostate is immobilized, the patient motion during treatment is more important in that the patient seldom moves and the movement is abrupt. Therefore, the time scales needed to determine the real‐time position are less stringent and may be on the order of 5 s to 10 s or greater, depending upon the dose rate and delivery method. For treatments involving both prostate and patient immobilization, localization is determined mainly at treatment setup. Real‐time tracking in this case would be less useful than in the previous conditions. However, it would track potential position drift or immobilization failure during treatment. The time scales for these events would be greater than 10 s.

Localization accuracy for real‐time localization is another design constraint needed for consideration. Currently, the treatment‐planning margins for setup are large. Estimates for these are between 5 mm and 10 mm, depending upon the direction. In general, the localization uncertainty should be on the order of imaging and drawing uncertainties, which is approximately 2 mm, for dose escalation. This value may be debated depending upon the immobilization and treatment method. If the immobilization system prevents prostate and skeletal dynamic motion and the planning margins are small, the system benefits from using a very accurate position measurement. In this case, patient setup accuracy is more important than dynamic localization. Treatments involving no prostate immobilization and dynamic corrective strategy may have larger planning margins and require less localization accuracy. From estimates in terms of localization accuracy and data acquisition time intervals, the accuracy in the 3D position should be approximately 2 mm, and coordinate updates should be on the order of seconds to accommodate the more rigorous requirements.

In addition to time and accuracy constraints, the physical size of the transmitter must be small. This constraint mainly affects the data acquisition time interval due to the small current loop used for power transfer. From Eq. (12), the voltage increases as the mutual inductance and frequency increase. This implies that the dipole antenna can be operated at a higher frequency to increase power transfer and thus decrease the charge‐up time. However, there is a frequency limit imposed on this by attenuation from intervening tissue. A better solution to the problem is to increase the mutual inductance between the two circuits. From Eq. (8), the mutual inductance increases proportional to the area of the implant inductor loop and the associated magnetic flux. Increasing the area will increase the mutual inductance by $r^{2}$. For our estimates, the chosen radius value is ~2mm to 3 mm. Increasing this to 4 mm to 5 mm can increase the flux by an order of magnitude, but the values chosen are closer to the maximum extent allowed for clinical application. The other parameter that can be varied is the magnetic induction field within the implant transmitter. The magnetic flux within the current loop can be modified by the external magnetic field from the dipole antenna or the magnetic permeability. By using a ferrite core within the current loop, the relative permeability will increase by about 3 orders of magnitude. Depending upon the inductor material used, the permeability may provide an additional increase by up to several orders of magnitude. To provide a conservative estimate of the mutual induction without using special materials, the relative permeability value of 2000 was chosen for the calculations. An increase in this value would decrease the charge‐up time.

The mutual inductance can be increased by increasing the magnetic field strength of the dipole antenna relative to the dipole current, orienting the dipole loop parallel with the implant current loop and decreasing the distance between the two coils. The field strength can be increased slightly, by factors of 2 to 10, by increasing the number of current loops. Also, an inductor core may be added to the configuration. The distance and orientation between the dipole antenna and transmitter are determined by the patient size and clinical environment. This part of the design is constrained by the patient size, the couch, and the accelerator. There are a number of possible dipole configurations that can be implemented. One possibility is aligning the magnetic dipole moment along the couch longitude direction. This basic configuration maximizes the relative orientation and decreases the distance between the two current loops. The dipole current loop can be designed to move freely across the couch and be fixed at a minimal distance from the transmitter. Since it is a thin wire loop, radiation can be delivered through it with minimal attenuation and interference. In addition, the shape and size can be designed to avoid collisions with the accelerator without much effort. With respect to the implant orientation, the relative orientation of the two magnetic moments is not defined and can vary. For a prostate implant, the magnetic moments of the two current loops will be relatively parallel but not exactly. This reduces the field strength, but it is not expected to be more than by a factor of 2 to 4. Adding another antenna placed perpendicular to the first would reduce the orientation effects and contribute to the mutual inductance. Typical distance values for this type of configuration are approximately 50 cm or less. The mutual inductance values used in the previous calculations were estimated using good coupling at a distance of 0.5 m where the magnetic moments are parallel. In general, this is not true but illustrates the importance of the dipole antenna design and mutual inductance for power transfer. Using more dipole antennas will increase the coupling and power transfer to the implant inductor. If only one dipole is used, the mutual inductance my decrease by up to an order of magnitude.

With low values in mutual inductance predicted, duplex operating modes are difficult to establish. Operating at resonant frequency can potentially increase the supply voltage by two orders of magnitude, but that increase is just at the limit needed for operating most chips. With fluctuations in the mutual inductance, the transmitted signal may be erratic. If the circuit is designed to simply ring in response to the alternating induction field, the calculations show that the resultant magnetic field strength will be at the limit of detection, ~1pT, using SQUID magnetometers at the desired accuracy of 2 mm. These methods for generating a continuous signal for detection are near the limit of operation given the constraints and estimates for this system.

Instead of generating a continuous signal, an alternating signal from the transmitter can be pulsed at varying times with time intervals on the order of 1 s. This method allows the transmitter inductor current to be integrated to create a residual charge that can act as a supply voltage and then discharged through the necessary circuit components to create an alternating current. With the previous estimates of $\sim 10^{-5}\;C$ integrated over 1 s, the signal current can be $10^{-2}$ A or greater, depending upon the method chosen for pulse shaping over a time interval of $10^{-3}\;s$. Correspondingly, at 1 m the magnetic induction field along the coil axis is $\sim 10^{-7}\;T$, which is easily measured. For 2 mm accuracy, the field detection level is roughly 0.1 nT, which can be measured using a SQUID magnetometer. These estimates are based upon integrating the current over one time interval and then discharging over a much smaller time.

The additional advantage of using a sequential method to integrate the inductor charge is that the charge collection can occur before treatment. It is possible to charge the transmitter circuit to much larger values, $\sim 10^{-2}\;C$, by integrating over several minutes instead of 1 s. The resultant charge will then create the necessary microchip operating voltage. The magnetic field can easily be created using all inductively generated current during the real‐time charging.

Even with the lower estimates of the magnetic field strength along the implant coil axis, SQUID magnetometers will be able to detect the signal generated in a simple sequential charging method. The problem will be with off‐axis values where the values will be smaller. Even though these values will be near the detection limit, it is better to maximize the field strength by multiple dipole sources and pretreatment charging. The advantages of using SQUID magnetometers are due to the low detection level and the small size. The ability to detect small induction fields allows the detectors to be placed at more convenient locations in the treatment room without constraining the devices to be located within the field or very close to the patient. The small size reduces gradient effects and increases accuracy. Using conventional magnetometers will decrease design flexibility and overall accuracy.

## V. CONCLUSION

We investigated localizing the prostate for real‐time radiotherapy treatment using the magnetic induction field as the source for calculating the position. This approach demonstrates that a static permanent magnet lacks the field strength for detection in a clinical environment. Thus, the method requires oscillating the field strength in order to be detected from the background noise. We examined an inductively coupled transmitter as a passively charged source to avoid complications using batteries as part of the implant design. To charge the transmitter, a dipole antenna was considered as the power source and SQUID magnetometers were assumed for establishing detection limits for the overall design.

From the fundamental analysis employed in this paper, we determined the basic operating characteristics needed for successful detection, including the dipole as well as the transmitter. Due to the magnetic field attenuation within body tissue, the operating frequency of the transmitter will generate larger detection signals in the low‐frequency range for frequencies less than approximately 200 kHz. Similarly, operating the dipole source at lower frequencies increases induction even though the induction current is reduced at lower frequencies. Operating at low frequencies has other effects as well, including reducing the problem to a near field solution and enabling simple circuit analysis for the problem. With this, the major limitation identified for operating in real time is the mutual inductance between the transmitter and the dipole antenna.

Two modes for charging the transmitter were examined, continuous and sequential. It was demonstrated that the sequential mode is better due to the overall low mutual inductance and that the mutual inductance continually changes as the patient moves. Operating at the resonant frequency in the continuous mode increases the supply voltage and decreases the required induction field but not to the extent that is needed for these conditions, where the magnetic flux is very small (the size constraint placed on the implant requires that the loop radius be small, which limits the flux).

With regard to the dipole antenna design, the loop radius is approximately equal to the distance from the dipole to the transmitter. This implies that the location of the dipole should not be neglected from a practical design consideration. However, since the wire diameter is small, the loop can be in the radiation path as long as there are no collision paths with respect to the couch or gantry. Furthermore, the number of dipole sources can be increased to enhance the mutual inductance and improve both the design flexibility and charge time.

From the analysis, the sequential operating mode is a feasible method for generating detectable magnetic induction field strengths within approximately one second. This allows for uncertainties in mutual inductance between the transmitter and the dipole source: the total charge is specified, and the time is allowed to vary. In the simplest configuration, the charge time can be on the order of seconds and can vary due to changes in orientation. Including additional induction field sources to eliminate orientation problems and a pretreatment charge can easily reduce the charge time between transmissions.

## ACKNOWLEDGMENT

This work was supported in part by The Methodist Hospital Foundation and the Department of the Army, US Army Medical Research Grant No. W81XWH‐04‐1‐0054.

## Supporting information

Supplementary Material

## References

[1] Crook JM , Raymond Y , Salhani D , et al. Prostate motion during standard radiotherapy as assessed by fiducial markers. Radiother Oncol. 1995;37:35–42. 8539455 10.1016/0167-8140(95)01613-l

[2] Balter JM , Sandler HM , Lam KL , et al. Measurement of prostate movement over the course of routine radiotherapy using implanted markers. Int J Radiat Oncol Biol Phys. 1995;31:113–118. 7995741 10.1016/0360-3016(94)00382-U

[3] Balter JM , Lam KL , Sandler HM , et al. Automated localization of the prostate at the time of treatment using implanted radiopaque markers: Technical feasibility. Int J Radiat Oncol Biol Phys. 1995;33:1281–1286. 7493853 10.1016/0360-3016(95)02083-7

[4] van Herk M , Bruce A , Kroes AP , et al. Quantification of organ motion during conformal radiotherapy of the prostate by three dimensional image registration. Int J Radiat Oncol Biol Phys. 1995;33:1311–1320. 7493856 10.1016/0360-3016(95)00116-6

[5] Balter JM , Ten Haken RH , Lawrence TS , et al. Uncertainties in CT‐based radiation therapy treatment planning associated with patient breathing. Int J Radiat Oncol Biol Phys. 1996;36:167–174. 8823272 10.1016/s0360-3016(96)00275-1

[6] Melian E , Mageras G , Fuks GS , et al. Variation in prostate position quantification and implications for three‐dimensional conformal treatment planning. Int J Radiat Oncol Biol Phys. 1997;38:73–81. 9212007 10.1016/s0360-3016(97)00221-6

[7] Antolak JA , Rosen II , Childress CH , et al. Prostate target volume variations during a course of radiotherapy. Int J Radiat Oncol Biol Phys. 1998;42:661–672. 9806528 10.1016/s0360-3016(98)00248-x

[8] Kim J , Fessler JA , Lam KA , et al. A feasibility study of mutual information based setup error estimation for radiotherapy. Med Phys. 2001;28(12):2507–2517. 11797954 10.1118/1.1420395

[9] Alasti H , Petric MP , Canton CA , et al. Portal imaging for evaluation of daily on‐line setup errors and off‐line organ motion during conformal irradiation of carcinoma of the prostate. Int J Radiat Oncol Biol Phys. 2001; 49(3):869–884. 11172971 10.1016/s0360-3016(00)01446-2

[10] Malone S , Szanto J , Perry G , et al. A prospective comparison of three systems of patient immobilization for prostate radiotherapy. Int J Radiat Oncol Biol Phys. 2000;48(3):657–665. 11020561 10.1016/s0360-3016(00)00682-9

[11] Hanley J , Lumley MA , Mageras GS , et al. Measurement of patient positioning errors in three‐dimensional conformal radiotherapy of the prostate. Int J Radiat Oncol Biol Phys. 1997;37:435–444. 9069319 10.1016/s0360-3016(96)00526-3

[12] Beard CJ , Kijewski P , Bussiaere M , et al. Analysis of prostate and seminal vesicle motion: Implications for treatment planning. Int J Radiat Oncol Biol Phys. 1996;34:451–458. 8567348 10.1016/0360-3016(95)02081-0

[13] Schewe JE , Balter JM , Lam KL , et al. Measurement of patient setup errors using port films and a computer‐aided graphical alignment tool. Med Dosim. 1996;21(2):97–104. 8807610 10.1016/0958-3947(96)00022-2

[14] Meertens H , van Herk M , Bijold J , et al. First clinical experience with a newly developed electronic portal imaging device. Int J Radiat Oncol Biol Phys. 1990;18(5):1173–1181. 2347724 10.1016/0360-3016(90)90455-s

[15] Rudat V , Schraube P , Oetzel D , et al. Combined error of patient positioning variability and prostate motion uncertainty in 3D conformal radiotherapy of localized prostate cancer. Int J Radiat Oncol Biol Phys. 1996;35: 1027–1034. 8751412 10.1016/0360-3016(96)00204-0

[16] Vigneault E , Pouliot J , Laverdiere J , et al. Electronic portal imaging device detection of radio‐opaque markers for the evaluation of prostate position during megavoltage irradiation: A clinical study. Int J Radiat Oncol Biol Phys. 1997;37:205–212. 9054897 10.1016/s0360-3016(96)00341-0

[17] Lattanzi J , McNeeley S , Pinover W , et al. Daily CT localization for correcting portal errors in the treatment of prostate cancer. Int J Radiat Oncol Biol Phys. 1998;41:1079–1086. 9719118 10.1016/s0360-3016(98)00156-4

[18] McGary JE , Grant W III . A clinical evaluation of setup errors for a prostate immobilization system. J Appl Clin Med Phys 2000;1(4):138–147. 11674829 10.1120/jacmp.v1i4.2635 PMC5726153

[19] McGary JE , Teh BS , Butler EB , et al. Prostate immobilization using a rectal balloon. J Appl Clin Med Phys. 2002;3(1):6–11. 11817999 10.1120/jacmp.v3i1.2590 PMC5724550

[20] Teh BS , Mai WY , Uhl B , et al. Intensity‐modulated radiation therapy (IMRT) for prostate cancer with the use of a rectal balloon for prostate immobilization: Acute toxicity and dose‐volume analysis. Int J Radiat Oncol Biol Phys. 2001;49(3):705–712. 11172952 10.1016/s0360-3016(00)01428-0

[21] Teh BS , Mai WY , Grant W , et al. Intensity modulated radiotherapy (IMRT) decreases treatment‐related morbidity and potentially enhances tumor control. Cancer Invest. 2002;20(4):437–451. 12094538 10.1081/cnv-120002143

[22] Boyer AL , Antonuk L , Fenster A , et al. A review of electronic portal imaging devices (EPIDs). Med Phys. 1992; 19(1):1–16. 1620036 10.1118/1.596878

[23] De Neve W , Van den Heuvel F , Coghe M , et al. Interactive use of on‐line portal imaging in pelvic radiation. Int J Radiat Oncol Biol Phys. 1993;25: 517–524. 8436530 10.1016/0360-3016(93)90075-7

[24] Gilhuijs K , Touw A , van Herk M , et al. Optimization of automatic portal image analysis. Med Phys. 1995;22: 1089–1099. 7565383 10.1118/1.597610

[25] Dong L , Shiu A , Boyer A , et al. Verification of radiosurgery target point alignment with an electronic portal imaging device (EPID). Med Phys. 1997;24(2):263–267. 9048367 10.1118/1.598070

[26] Bergstrom P , Lofroth PO , Windmark A . High precision conformal radiotherapy (HPCRT) of prostate cancer—a new technique for exact positioning of the prostate at the time of treatment. Int J Radiat Oncol Biol Phys. 1998; 42:305–311. 9788408 10.1016/s0360-3016(98)00229-6

[27] Herman MG , Balter JM , Jaffray DA , et al. Clinical use of electronic portal imaging: Report of AAPM Radiation Therapy Committee Task Group 58. Med Phys. 2001;28(5):712–737. 11393467 10.1118/1.1368128

[28] el‐Gayed AA , Bel A , Vijbrief R , et al. Time trend of patient setup deviations during pelvic irradiation using electronic portal imaging. Radiother Oncol. 1993;26(2):162–171. 8465017 10.1016/0167-8140(93)90098-s

[29] Greer PB , Mortensen TM , Jose CC , et al. Comparison of two methods for anterior‐posterior isocenter localization in pelvic radiotherapy using electronic portal imaging. Int J Radiat Oncol Biol Phys. 1998;41(5):1193–1199. 9719132 10.1016/s0360-3016(98)00160-6

[30] Hornick DC , Litzenberg DW , Lam KL , et al. A tilt and roll device for automated correction of rotational setup errors. Med Phys. 1998;25(9):1739–1740. 9775381 10.1118/1.598355

[31] Litzenberg DW , Balter JM , Hornick DC , et al. A mathematical model for correcting patient setup errors using a tilt and roll device. Med Phys. 1999;26(12):2586–2588. 10619243 10.1118/1.598797

[32] Bergstrom P , Lofroth PO , Windmark A . High precision conformal radiotherapy (HPCRT) of prostate cancer—a new technique for exact positioning of the prostate at the time of treatment. Int J Radiat Oncol Biol Phys. 1998; 42:305–311. 9788408 10.1016/s0360-3016(98)00229-6

[33] Ezz AM , Munro P , Porter AT , et al. Daily monitoring and correction of radiation field placement using a video‐based portal imaging system: A pilot study. Int J Radiat Oncol Biol Phys. 1992;22:159–165. 1727113 10.1016/0360-3016(92)90995-t

[34] Lattanzi J , McNeeley S , et al. A comparison of daily CT localization to a daily ultrasound‐based system in prostate cancer. [see comments.]. Int J Radiat Oncol Biol Phys. 1999;43(4):719–725. 10098426 10.1016/s0360-3016(98)00496-9

[35] Mohan DS , Kupelian PA , et al. Short‐course intensity‐modulated radiotherapy for localized prostate cancer with daily transabdominal ultrasound localization of the prostate gland. [see comments.] Int J Radiat Oncol Biol Phys. 2000;46(3):575–580. 10701736 10.1016/s0360-3016(99)00454-x

[36] Vigneault E , Pouliot J , et al. Electronic portal imaging device detection of radio‐opaque markers for the evaluation of prostate position during megavoltage irradiation: A clinical study. Int J Radiat Oncol Biol Phys. 1997; 37(1):205–212. 9054897 10.1016/s0360-3016(96)00341-0

[37] Russell K , Skrumeda L , Gisselberg M , et al. Biocompatibility of a wireless electromagnetic transponder permanent implant for accurate localization and continuous tracking of tumor targets. Int J Radiat Oncol Biol Phys. 2003;57(2 Suppl):S396–S397.

[38] Lorain P , Corson D . Electromagnetic fields and waves. W.H. Freeman and Company; 1970.

[39] Lo YT , Lee SW . Antenna handbook, vol. III Kluwer Academic Publishers; 1993.

