# Supplementary material for: Theoretical foundation for real‐time prostate localization using an inductively coupled transmitter and a superconducting quantum interference device (SQUID) magnetometer system
Source: J Appl Clin Med Phys. 2004 Nov 24;5(4):29–45. doi: 10.1120/jacmp.v5i4.2021 (PMC5723525; doi:10.1120/jacmp.v5i4.2021)
Supplement: Supplementary file 1 — Supplementary Material [file ACM2-5-029-s001.doc]

Theoretical foundation for real-time prostate localization using an inductively coupled transmitter and a superconducting quantum interference device (SQUID) magnetometer system

# John. E. McGary

Department of Radiology, Baylor College of Medicine, Houston, Texas 77030

[*mailto:jmcgary@earthlink.net*](mailto:jmcgary@earthlink.net)
